# Metabolites of 2,3-diketogulonate delay peroxidase action and induce non-enzymic H_2_O_2_ generation: Potential roles in the plant cell wall

**DOI:** 10.1016/j.abb.2017.03.006

**Published:** 2017-04-15

**Authors:** Anna Kärkönen, Rebecca A. Dewhirst, C. Logan Mackay, Stephen C. Fry

**Affiliations:** a Department of Agricultural Sciences, Viikki Plant Science Center, University of Helsinki, Finland; b The Edinburgh Cell Wall Group, Institute of Molecular Plant Sciences, The University of Edinburgh, Edinburgh EH9 3BF, UK; c EastCHEM School of Chemistry, The University of Edinburgh, Edinburgh EH9 3FJ, UK

**Keywords:** Ascorbate, Dehydroascorbic acid, Diketogulonate, Apoplast, Peroxidase, Hydrogen peroxide, Hydroxyl radical, Plant cell wall, AAO, ascorbate oxidase, ABTS, 2,2′-azino-bis(3-ethylbenzthiazoline-6-sulphonic acid, DHA, dehydroascorbate, DKG, 2,3-diketo-l-gulonic acid, 2,3-enediol-DKGL, the 2,3-enediol form of 2,3-diketogulono-δ-lactone, 3,4-enediol-DKGL, the 3,4-enediol form of 2,3-diketogulono-δ-lactone, ROS, reactive oxygen species, XO, xylenol orange

## Abstract

A proportion of the plant's l-ascorbate (vitamin C) occurs in the apoplast, where it and its metabolites may act as pro-oxidants and anti-oxidants. One ascorbate metabolite is 2,3-diketogulonate (DKG), preparations of which can non-enzymically generate H_2_O_2_ and delay peroxidase action on aromatic substrates. As DKG itself generates several by-products, we characterised these and their ability to generate H_2_O_2_ and delay peroxidase action.

DKG preparations rapidly produced a by-product, compound (**1**), with *λ*_max_ 271 and 251 nm at neutral and acidic pH respectively. On HPLC, (**1**) co-eluted with the major H_2_O_2_-generating and peroxidase-delaying principle. Compound (**1**) was slowly destroyed by ascorbate oxidase, and was less stable at pH 6 than at pH 1. Electrophoresis of an HPLC-enriched preparation of (**1**) suggested a strongly acidic (p*K*_a_ ≈ 2.3) compound. Mass spectrometry suggested that un-ionised (**1**) has the formula C_6_H_6_O_5_, i.e. it is a reduction product of DKG (C_6_H_8_O_7_).

In conclusion, compound (**1**) is the major H_2_O_2_-generating, peroxidase-delaying principle formed non-enzymically from DKG in the pathway ascorbate → dehydroascorbic acid → DKG → (**1**). We hypothesise that (**1**) generates apoplastic H_2_O_2_ (and consequently hydroxyl radicals) and delays cell-wall crosslinking — both these effects favouring wall loosening, and possibly playing a role in pathogen defence.

## Highlights

• Diketogulonate (DKG) is an ascorbate metabolite formed in plant cell walls.
• DKG byproduct(s) nonenzymically generate H_2_O_2_ but delay peroxidase action *in vitro*.
• The major byproduct is oxidised by ascorbate oxidase more slowly than ascorbate.
• Its UV spectrum resembles ascorbate's; it may be a DKG reduction product, C_6_H_6_O_5_.
• The apoplastic byproduct of DKG may affect cell expansion, fruit softening or defence.

## 1 Introduction

l-Ascorbic acid (C_6_H_8_O_6_; vitamin C) is an important redox compound in all plants and animals. In plants, it is synthesised in the protoplast, and a proportion of it is released into the apoplast (aqueous solution that permeates the cell wall) [37], [11], where some of it is enzymically and non-enzymically oxidised by O_2_ to form monodehydroascorbate, which rapidly disproportionates into ascorbate and dehydro-l-ascorbic acid (DHA; C_6_H_6_O_6_). Ascorbate oxidase is well established to be a wall-localised enzyme capable of modulating the ascorbate:DHA ratio [43]. Apoplastic ascorbate and its downstream metabolites have been widely discussed as important players in protecting the plant against environmental oxidative stresses, especially ultraviolet irradiation, atmospheric ozone pollution and pathogen challenge [5], [40], [10], [41], [44], [4], [47], [53]. Apoplastic ascorbate metabolites have also been proposed to serve roles in modulating the cell wall's biophysical properties, especially influencing the softening of ripening fruits and the extensibility of the primary wall [12], [25], [15], [9], [1].

DHA is unstable in neutral aqueous solutions and is easily de-lactonised to 2,3-diketo-l-gulonic acid (DKG; C_6_H_8_O_7_) which itself converts non-enzymically to several further degradation products depending on the incubation conditions [51], [26], [8], [46], [35]. In the apoplast of cultured rose cells, a portion of the DHA is oxidised to oxalate and l-threonate (and esters thereof), some of these reactions being proposed to generate H_2_O_2_, and a further portion of the DHA is hydrolysed to DKG [17], [18]. The balance between these two pathways (oxidation:hydrolysis ratio of DHA) is dictated by the severity of the ambient oxidising conditions [39], [38]. Some ascorbate degradation products are of interest in cell-wall physiology and in pathogen defence because of their unique redox properties.

DKG is a highly unstable compound, readily forming a wide range of by-products. Some of the many reported ascorbate degradation products, probably formed via DKG, include 2,3-enediol-DKGL (C_6_H_6_O_6_; the 2,3-enediol form of 2,3-diketo-gulono-δ-lactone), 3,4-enediol-DKGL (also C_6_H_6_O_6_; the 3,4-enediol form of 2,3-diketo-gulono-δ-lactone) and l**-**erythroascorbic acid (C_5_H_6_O_5_), have reducing activity and might function as reducing agents in a similar way to ascorbate *in vivo* [48], [26], [27], [35]. The pathway from DKG to erythroascorbate was suggested to proceed via 2,3-enediol-l-lyxose, with O_2_ (or DHA if present) serving as oxidant in the conversion of 2,3-enediol-l-lyxose to erythroascorbate [19]. However, the physiological significance of erythroascorbate formation from DKG may be limited, as the process was only rapid in the presence of cyanide, 0.5 M phosphate, and a pH of 8.

De-lactonisation of DHA to DKG is often considered to be irreversible [35]. However, some formation of DHA by re-lactonisation of DKG has been demonstrated, especially at lower pH values [31]. DHA can be reduced to ascorbate when a suitable reductant is present; 3,4-enediol-DKGL formed from DKG was considered to be this reductant [48]. Indeed, the concentration of 3,4-enediol-DKGL rose to 10% of that of DKG after 30 min incubation of DKG at neutral pH in a nitrogen atmosphere [48].

DKG has also been reported to undergo decarboxylation to l-xylosulose (= l-xylosone) [55], [32], [19], from which erythroascorbate may be formed [21]. l-Xylosulose may also give rise to several strongly acidic redox compounds e.g. 2-furoic acid and the de-lactonised form of 5-methyl-3,4-dihydroxytetrone, although most of these were only formed under highly unphysiological conditions such as 0.5 M H_2_SO_4_ at 90 °C [26]. Non-acidic dioxo products may be formed from DKG after the loss of oxalate by hydrolysis to form l-erythrulose and subsequently 3-deoxy-l-threosulose (= 3-deoxy-l-threosone) or after the loss of oxalate by an oxidative pathway to form l-threosulose — all of which are compounds discussed as being of relevance to the ageing of animal lens proteins [34]. However, we are not aware of any of these particular dioxo products being reported to delay peroxidase action or to reduce O_2_ to H_2_O_2_.

Additional products of DKG degradation, formed in the plant apoplast, include compounds ‘**C**’ and ‘**E**’ [17], provisionally identified [39] as **C** = 2-carboxy-l-xylonolactone plus 2-carboxy-l-lyxonolactone and **E** = their de-lactonised product; **C** and **E** are interconvertible, but otherwise relatively stable both *in vivo* and *in vitro*.

‘DKG’ preparations have biologically interesting redox properties, probably due to the formation of DKG degradation products. An ene–diol group or some other functional group that is as easily oxidisable as an ene–diol is thought to be a common feature of the ascorbate degradation products having reducing properties [51]. For example, ‘DKG’ (≥120 μM) has been reported to be an anti-oxidant against the oxidative modification of yolk lipoprotein in a copper-containing solution, whereas it has a pro-oxidative effect at lower concentrations (≤75 μM; [29]). Furthermore, ‘DKG’ at micromolar concentrations delays copper-induced oxidative formation of conjugated dienes in yolk lipoprotein, the lag time lengthening with increasing ‘DKG’ concentrations [29]. Since DKG itself has no reducing activity, it was suggested that 3,4-enediol-DKGL, the most prevalent breakdown product detected, was responsible for the anti-oxidative effect [29]. 2,3-Enediol-DKGL was also present and possibly contributed to the anti-oxidative function. Likewise, [42] hypothesised that the protective effect of DHA on copper-induced oxidative modification of human low-density lipoprotein was due to stable modification of the protein by DHA or its breakdown product(s).

‘DKG’ has previously been observed to accelerate the peroxidation of linoleic acid in neutral but not in slightly acidic solutions [49]. A superoxide-scavenging agent, Tiron, suppressed linoleate peroxidation whereas catalase had no inhibitory effect, suggesting that superoxide was the reactive oxygen species (ROS) generated during incubation with DKG. Although H_2_O_2_ was the ROS detected in the present study, the possibility remains that superoxide was the original ROS generated, forming H_2_O_2_ by dismutation.

Ascorbate induces clear non-enzymic H_2_O_2_ generation when added into a solution containing a transition metal [7], [12]. Also DHA, and especially a DKG preparation prepared from commercial DHA, led to H_2_O_2_ generation when added into a solution containing a trace of copper ions [22]. To detect the H_2_O_2_ generated after DKG addition we used two separate assays: the xylenol orange (XO) assay [16], [3] and an indirect peroxidase activity assay in which *o*-dianisidine was used as a peroxidase substrate. Differences in the results obtained by these two methods led to the discovery that the DKG preparation contained a compound that inhibits peroxidase activity. Since the DKG preparation contained several breakdown products, as observed by paper electrophoresis followed by silver staining (Fig. 9 in Ref. [22], we wanted to resolve which of these was the major active component inducing non-enzymic H_2_O_2_ generation and delaying peroxidase action.

## 2 Materials and methods

### 2.1 Chemicals

Ascorbate oxidase (AAO), 2,2′-azino-bis(3-ethylbenzthiazoline-6-sulphonic acid) (ABTS), catalase, dehydroascorbic acid and dehydro-l-ascorbic acid dimer, *o*-dianisidine dihydrochloride and horseradish peroxidase type II were obtained from Sigma-Aldrich. AAO was dissolved as a stock at 1000 U ml^−1^ in 50 mM succinate (Na^+^) buffer, pH 5.6, supplemented with 0.05% bovine serum albumin. Peroxidase was dissolved (1 μg μl^−1^) and further diluted in the same buffer.

DKG was prepared from the commercial DHA by alkali treatment [56]. A stock of DHA (50 mM) was prepared in water (it took at least 30 min to dissolve DHA). A slight molar excess of NaOH (1.3 × ) was added and the mixture incubated at 20 °C for 6 min. Routinely, the hydrolysis was then stopped with 1 M l-tartaric acid and the pH was checked by pH paper (∼3.5–4.0). However, for samples to be fractionated by HPLC, hydrolysis was stopped with 1 M H_2_SO_4_ to a final pH of ∼1 or ∼6. Freshly-made DHA and DKG solutions were stored on ice before the assays.

DKG was prepared also by an iodate method [20]. A solution of ascorbic acid (0.12 M) was incubated with potassium iodate (0.36 M) for 5 min. KOH (1 M) was then added dropwise until the solution became colourless. Cold ethanol (8 vol, −20 °C) was added, and the precipitated DKG was vacuum filtered, rinsed in 70% ethanol, dried and stored at −80 °C.

### 2.2 In-vitro peroxidase activity assays

The effects of various ascorbate breakdown products on peroxidase activity were tested *in vitro*. DHA and DKG stock solutions were freshly prepared and added at various concentrations to a reaction mixture (total volume 1.0 ml) that contained either 550 μM ABTS or 800 μM *o*-dianisidine, and 250 or 500 μM H_2_O_2_, 3.13 or 6.25 ng/ml horseradish peroxidase type II and the compound of interest, in 44 mM succinate (Na^+^) buffer, pH 5.6. [All concentrations quoted are final, in the complete reaction mixture, unless otherwise stated.] The reaction was initiated by the addition of the enzyme and followed at 420 and 405 nm for ABTS and *o*-dianisidine respectively. When an ascorbate oxidase (AAO) pre-treatment was included, the compound of interest (∼0.5 mM) was pre-incubated in 4 U/ml AAO and 44 mM succinate (Na^+^) buffer, pH 5.6, for 10, 15 or 60 min at 20 °C before addition of the other assay components.

### 2.3 Search for the active component(s) in the DKG preparation that stimulates H_2_O_2_ production and inhibits peroxidases

As the DKG preparation contained several compounds in addition to DKG (Fig. 9 in Ref. [22], these ‘metabolites’ were separated by preparative high-voltage paper electrophoresis at pH 2.0, 3.5 and 6.5 according to [14]. Each electrophoretogram was cut into strips, and the compounds were eluted from the paper in water, concentrated *in vacuo* (SpeedVac, Savant) and stored at −75 °C. The effect of eluted compounds on non-enzymic H_2_O_2_ production was tested *in vitro*: 10 μl of each fraction, supplemented with 1 μM CuSO_4_, was tested for H_2_O_2_ formation by the xylenol orange (XO) method [3]. The XO method detects hydroperoxides that oxidise Fe^2+^ in an acidic solution, and the amount of ferric product is measured as a XO complex [16]. Also the effect of each fraction on peroxidase activity *in vitro* was tested. To confirm the identity and stability of compounds used in the assays, we re-electrophoresed each fraction at the original pH, and stained the solutes with AgNO_3_ [13].

### 2.4 Search for the AAO-responsive ‘metabolite’ in the DKG preparation by HPLC

DKG [4.7 mM, in 45 mM succinate (Na^+^) buffer, pH 5.6] was treated with AAO (12 U/ml) or denatured AAO (10 min boiling) at 25 °C for 15 min with gentle mixing, then the enzymic reaction was terminated by addition of H_2_SO_4_ to pH ∼1. Reaction products, analysed by HPLC, were compared with those in an untreated DKG aliquot.

### 2.5 Semi-purification of the active compound(s) in DKG preparation by HPLC

HPLC was used to purify the compound(s) that inhibits peroxidases and generates H_2_O_2_ when added into 1 μM Cu^2+^. DKG preparations (∼46 mM, pH ∼1 and ∼6) were filtered (0.4 μm, Chromacol), and 40 μl was fractionated on a Phenomenex Rezex ROA column, run (0.5 ml min^−1^) at 35 °C, routinely with 47 mM H_2_SO_4_ as mobile phase. In some experiments, 13 mM TFA [0.1% (v/v)] was used when a volatile mobile phase was required. Degradation products were detected by UV absorbance at various wavelengths.

The major peak of cmpd (**1**) was collected and stored frozen prior to analysis. Mass spectrometry measurements were performed by electrospray on a 12T SolariX Fourier transform mass spectrometer (Bruker Daltonics) equipped with an infinity cell and operating in positive mode. Spectra were the sum of 20 mass analyses and collected with a data size of 4 Mword. Agilent tune mix was used for external calibration. Analysis was achieved with Data Analysis version 4.4 (Bruker Daltonics).

### 2.6 Analysis of redox properties of HPLC-fractionated metabolites

For preparative purposes, HPLC fractions (0.5 ml) were collected and used in peroxidase or H_2_O_2_ assays either immediately or after storage. In the latter case the fractions were frozen in liquid nitrogen before storage at −75 °C. Since ascorbate degradation products were eluted from the HPLC column in 47 mM H_2_SO_4_ (pH ∼1), the assays were modified as follows. The peroxidase activity assay mixture (1.0 ml) contained (added in the following order; final concentrations are given): 25 mM Na_2_-succinate, 37 mM succinate (Na^+^) buffer (pH 5.6), ≤225 μl of the HPLC fraction (if <225 μl, the remaining volume was added as 47 mM H_2_SO_4_), 550 μM ABTS, 250 μM H_2_O_2_, and 3.13 ng/ml peroxidase. When an AAO treatment was included, AAO (1 U/ml; active or boiled) was added after the HPLC fraction had been mixed with the succinate; then, after 10 min incubation at 20 °C, ABTS and H_2_O_2_ were added and the assay was started by addition of peroxidase.

The assay mixture for non-enzymic H_2_O_2_ generation contained (final volume 3.0 ml): 8.3 mM Na_2_-succinate and ≤225 μl of HPLC fraction (if <225 μl, the remaining volume was added as 47 mM H_2_SO_4_) and 1 μM CuSO_4_ (added last). When an AAO pre-treatment was included, the enzyme (0.7 U/ml) was added after the HPLC fraction had been mixed with the succinate, and the vials were incubated for 10 min on a shaker (100 rpm) at 20 °C before addition of CuSO_4_ (to 1 μM). As a control for the AAO treatment, the HPLC fraction was treated for 10 min with denatured AAO (10 min boiling). This treatment was also important to show whether incubation at an increased pH was enough to alleviate the effect of the compound, i.e. whether the compound was more labile at pH∼5 than at pH∼1.

H_2_O_2_ generated was measured by the XO assay [16], [3], [24]. Aliquots (100 μl) of the reaction mixture were sampled at time points and immediately added to 1 ml of XO mixture (containing 125 μM XO, 100 mM d-sorbitol, 250 μM FeSO_4_, 250 μM (NH_4_)_2_SO_4_ and 25 mM H_2_SO_4_). All sample-XO mixtures were incubated for 40 min at room temperature before measurement of *A*_560_ against a blank prepared with 100 μl solution containing 1 μM CuSO_4_ and 8.3 mM Na_2_-succinate + 1 ml of XO mixture. CuSO_4_ was observed not to interfere with the XO assay. A standard curve was prepared with different concentrations of H_2_O_2_ in 1 μM CuSO_4_. A dilution series of H_2_O_2_ was prepared in water and a portion of each solution was adjusted to 1 μM CuSO_4_ immediately before addition to XO reagent.

## 3 Results

### 3.1 Diketogulonate generates a by-product that reduces O_2_ to H_2_O_2_ and delays peroxidase action on model substrates

DKG, prepared by hydrolysis of DHA, exhibited a prominent peak of UV absorbance at pH 5.6 (*λ*_max_ 271–272 nm; [22]. In contrast, the DHA had negligible absorbance at this wavelength or at 265 nm (which is the *λ*_max_ of ascorbate, erythroascorbate and 3,4-enediol-DKGL in neutral solution; Table 1). Since freshly-made aqueous DKG is stated to have no strong absorption above 225 nm [36], the observed absorbance at 271–272 nm was probably attributable to unidentified DKG degradation products, the major one of which is here termed cmpd (**1**).

**Fig. 1:**
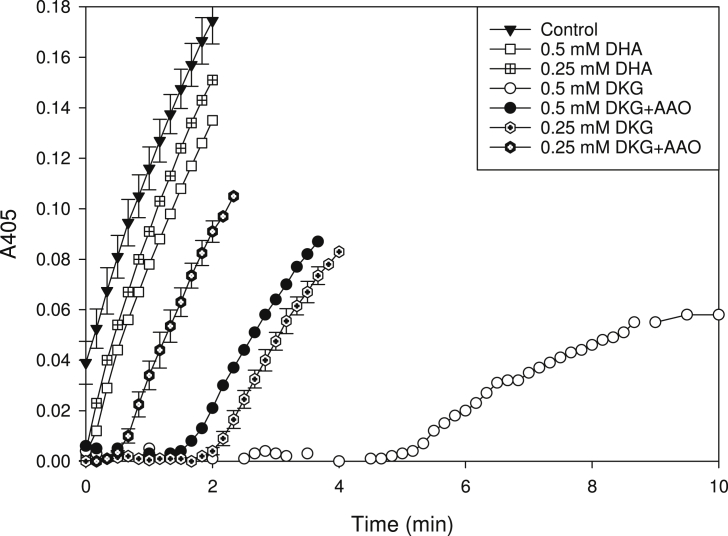
Effect of dehydroascorbate and a diketogulonate preparation on the peroxidase reaction with *o-*dianisidine as substrate. The effect of the DKG preparation pre-treated with AAO is also shown (+AAO). DHA, dehydroascorbic acid; DKG, diketogulonate.

Similar DKG solutions had been shown to reduce O_2_ to H_2_O_2_ non-enzymically in the presence of a trace of Cu^2+^ [22]. We now give evidence that this is due to the presence of cmpd (**1**). In addition, we have found that in *in-vitro* peroxidase assays, the DKG preparation caused a concentration-dependent lag, presumably also due to cmpd (**1**), before the oxidation of a model substrate, *o-*dianisidine, became visible (Fig. 1). After this lag, the reaction velocity was similar to (or, in the case of high ‘DKG’ concentrations, slightly slower than) that of the non-inhibited peroxidase, and the duration of the lag correlated with the amount of ‘DKG’ added. These effects, which seem unlikely to be due to DKG itself, are similar to those exerted by ascorbate [50]. DHA, on the contrary, showed little inhibition of peroxidase activity.

### 3.2 Ascorbate oxidase partially inactivates cmpd (**1**)

Since the UV spectrum of the DKG preparation (*λ*_max_ 271 nm at pH 5.6; Fig. 8 of [22] was reminiscent of that of ascorbate (*λ*_max_ 265 nm at pH 5.6), we explored the possible presence of ascorbate-like substances. Pre-treatment of the DKG preparation with ascorbate oxidase (AAO) diminished but did not abolish the lag period (Fig. 1). The *A*_271_ of the DKG preparation decreased slowly without any enzyme addition; addition of AAO accelerated this reaction (Fig. 8 of [22], but it took several minutes before the absorbance value reached a minimum, and even then some *A*_271_ persisted. In contrast, the *A*_265_ of a solution of pure ascorbate reaches zero a few seconds after AAO addition (Supplemental Fig. 1), confirming that cmpd (**1**) is not ascorbate. In a mixture of the DKG preparation and pure ascorbate, AAO caused a rapid decrease in *A*_265_ (due to ascorbate oxidation) followed by a slower decrease due to cmpd (**1**) oxidation (Supplemental Fig. 1); thus it cannot be argued that the DKG was inhibiting the AAO. Nevertheless, cmpd (**1**) appears to be ascorbate-related since AAO has a high specificity towards l-ascorbate and related compounds that have a lactone ring with an adjacent ene–diol group such as erythroascorbate [6].

### 3.3 High-voltage electrophoresis of the crude DKG preparation

As the alkali-generated DKG preparation contained several by-products [22], we attempted to separate these by electrophoresis and test them individually for peroxidase action delay (Supplemental Fig. 2) and non-enzymic H_2_O_2_ generation (data not shown). After electrophoresis at pH 2.0 (Supplemental Fig. 2a), only fraction 2 (containing neutral and weakly acidic material) exerted these effects, and only to a low degree. Thus the only active principle detected had clearly separated from DKG itself (a relatively strong acid, found in fractions 3 and 4). However, the total recovered zones had far less H_2_O_2_-generating and peroxidase-delaying capacity than the crude DKG that had been applied to the electrophoretogram.

After electrophoresis at pH 3.5 (Supplemental Fig. 2b) or 6.5 (Supplemental Fig. 2c), the only active principle detected was found to have co-migrated with DKG. This could indicate that (i) cmpd (**1**) co-migrated with DKG at these pH values, and/or (ii) the cmpd (**1**) originally present was degraded during the electrophoresis and subsequent elution but new cmpd (**1**) was formed from the eluted DKG itself.

### 3.4 HPLC of the DKG preparation

**Fig. 2:**
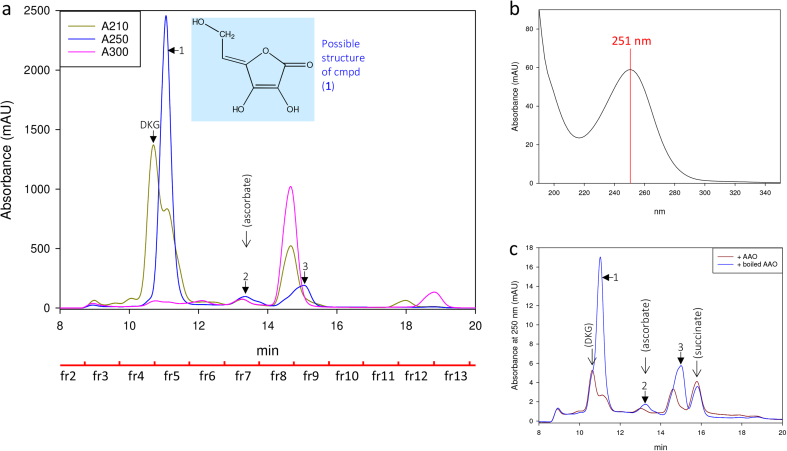
HPLC of diketogulonate and its by-products. (a) The DKG preparation was fractionated by HPLC with 47 mM H_2_SO_4_ as eluent, revealing several metabolites. The eluate was monitored simultaneously for absorbance at 210, 250 and 300 nm, and fractions (labelled fr1 to fr13) were collected. A possible structure for cmpd (**1**), based on its mass spectrum (Supplemental Fig. 5), is shown. (b) UV spectrum of the peak containing cmpd (1), eluting at 11.01 min. (c) Ascorbate oxidase (AAO; 12 U/ml), or denatured enzyme as a control, was applied to a new preparation of DKG for 15 min, then the products were fractionated as in (a). Absorbance at 250 nm is shown. Solid arrows, nomenclature of significant peaks; open arrows with names in brackets, expected elution positions of the named compounds.

On HPLC, freshly prepared crude DKG revealed several peaks of absorbance at 210 nm (*A*_210_ peaks), suggesting carboxylic acids, esters or lactones, and at least three *A*_250_ peaks (suggesting conjugated double-bonds; labelled **1**, **2** and **3** in Fig. 2). Cmpd (**1**) was eluted shortly after the DKG peak, only partially separated from it (retention times 11.02 and 10.64 min respectively; Fig. 2). We propose that cmpd (**1**) showed a pH-dependent absorbance shift, with *λ*_max_ 251 (Fig. 2b) and 271 nm at acidic and neutral pH respectively (the HPLC eluent was 47 mM H_2_SO_4_). This shift would mirror the behaviour of ascorbate and 3,4-enediol-DKGL, which have *λ*_max_ 245 and 265 nm in acidic and neutral solutions respectively [36], [48]. Cmpd (**1**) was again confirmed not to be ascorbate itself, as this elutes at 13.25 min in this system.

### 3.5 Ability of HPLC fractions to generate H_2_O_2_ and delay peroxidase action

**Fig. 3:**
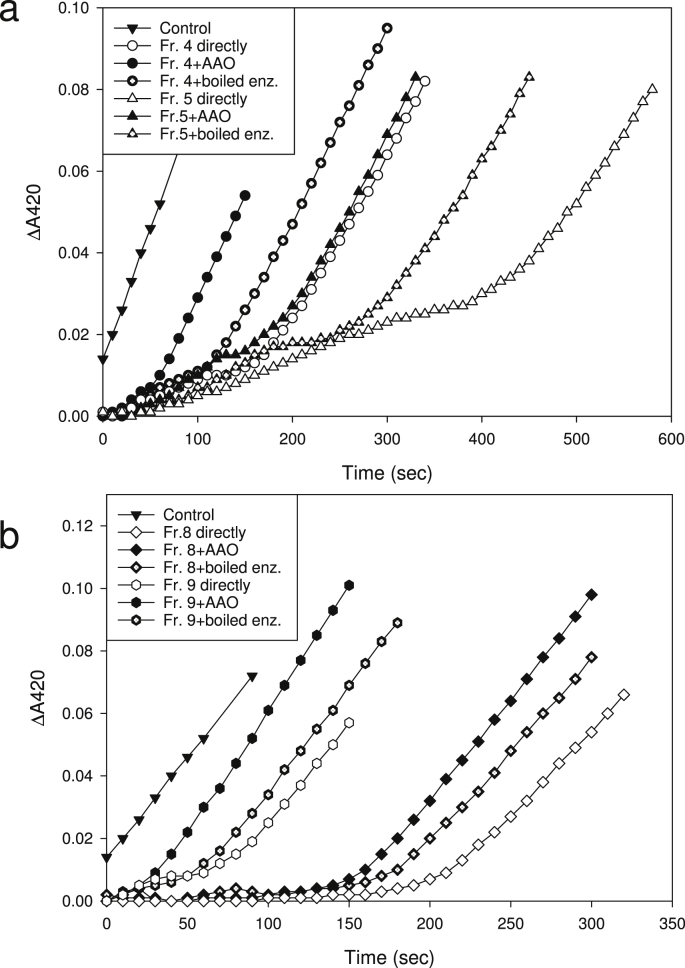
Selected HPLC fractions from a diketogulonate preparation delay peroxidase action. ABTS was used as peroxidase substrate. Volume of HPLC fraction added to the assay: 150 μl. The effect of pretreatment of the fractions with AAO (1 U/assay; +AAO) or with boiled AAO (+boiled enz) at pH ∼5.2–5.6 for 10 min before the peroxidase activity assay is also shown.

Fraction 5 (Fig. 2a), which contained the majority of cmpd (**1**), was the most effective fraction at delaying peroxidase action on a model substrate (ABTS; Fig. 3). Other delaying agents were also detected, e.g. in fractions 8 and 9. A moderate delaying effect was also observed in fraction 4, which contained most of the DKG (Fig. 2, Fig. 3). However, since 24% of cmpd (**1**) eluted in fraction 4, and because some of the DKG may be further degraded to cmpd (**1**) after elution from the column, we conclude that the peroxidase delaying agent in fraction 4 was cmpd (**1**), not the DKG itself.

Fractions 4 and 5 also caused non-enzymic H_2_O_2_ production in the presence of O_2_ and a trace of Cu^2+^, fraction 5 again being more effective (Supplemental Fig. 3). The other HPLC fractions tested, even those that caused a slight delay in peroxidase activity assays, did not generate H_2_O_2_.

### 3.6 Ascorbate oxidase diminishes the ability of HPLC fractions to delay peroxidase action and generate H_2_O_2_

AAO pre-treatment of the peroxidase-retarding HPLC fractions (4, 5, 8 and 9; Fig. 2a) diminished their ability to delay peroxidase action (Fig. 3). Treatment with heat-denatured AAO (i.e., ‘ageing’ the fractions at elevated pH (5.2–5.6) in the absence of active AAO) also slightly reduced the lag caused by fractions 4, 5, 8 and 9. However, AAO did not completely destroy the peroxidase delaying effects of any of these fractions, suggesting either that several agents were present in each fraction, only some of them being AAO-oxidisable, or that the AAO generated new products whose peroxidase-delaying properties were weaker than those of the initial compounds.

AAO-pretreatment of fractions 4 and 5 also diminished their ability to non-enzymically generate H_2_O_2_ (Supplemental Fig. 3).

### 3.7 UV-detectable compounds (**1**), (**2**) and (**3**) can be oxidised by ascorbate oxidase

Since AAO affected the HPLC fractions' effects on H_2_O_2_ generation and peroxidase action, we attempted to determine which UV-detectable compounds were affected by the AAO treatment. The crude DKG preparation was treated with AAO (active or denatured) for 15 min and re-run by HPLC. The most remarkable changes were observed in compounds absorbing at 250 nm (Fig. 2c). In particular, active AAO strongly diminished the cmpd (**1**) peak. A slight decrease was also noticed in the *A*_210_ of this peak (data not shown), suggesting that cmpd (**1**) is a carboxylic acid or ester as well as possessing conjugated double bonds. Compounds **2** and **3** also diminished (Fig. 2c; the peak eluting at 15.8 min is probably the succinate buffer).

### 3.8 Cmpd (**1**) is less stable at pH 6 than at pH 1

**Fig. 4:**
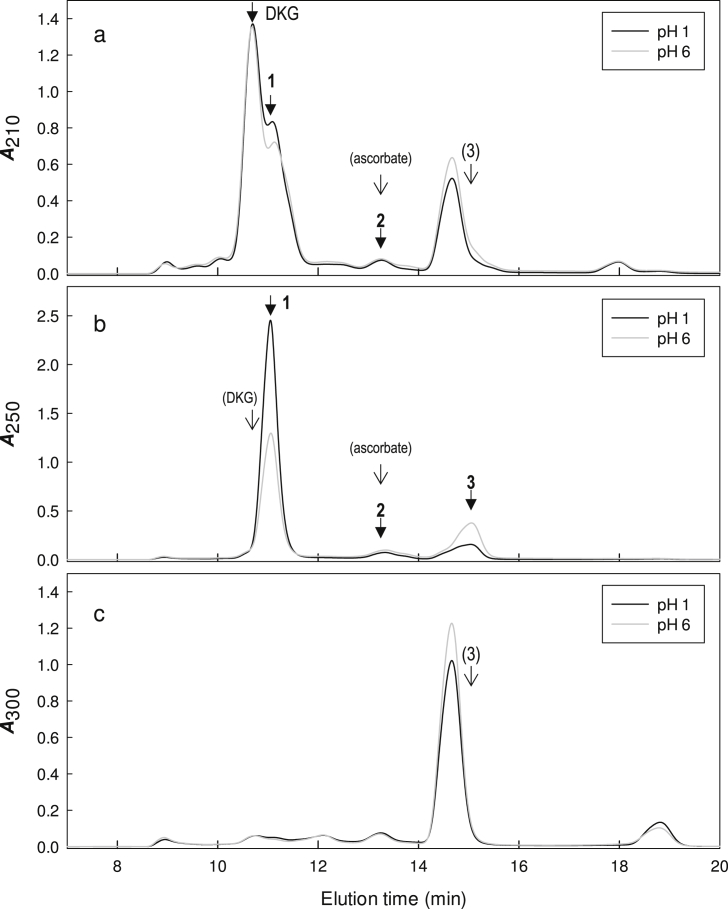
Effect of treatment at pH 1 or 6 on the HPLC profile of diketogulonate and its by-products. The DKG, prepared by NaOH treatment of DHA, was adjusted to pH 1 or pH 6 with H_2_SO_4_ and, after storage for 0.5–3.0 h at 0 °C, fractioned by HPLC. During each run, absorbances at (a) 210, (b) 250 and (c) 300 nm were simultaneously monitored. Solid arrows, nomenclature of significant peaks; open arrows with names in brackets, expected elution positions of the named compounds.

Since treatment even with denatured AAO (at pH ∼5.6) modified the HPLC profile (Fig. 2c) and the fractions' ability to delay peroxidase action (Fig. 3) and to promote H_2_O_2_ production (Supplemental Fig. 3), we tested the effect of pH on the stability of DKG and its by-products. For this work, DKG was prepared by alkaline hydrolysis of DHA and the reaction was stopped with H_2_SO_4_ either to pH 6 or to pH 1 (Fig. 4). DKG itself, detected at 210 nm, was almost unaffected by storage on ice for 0.5–3 h at pH 1 or 6 (Fig. 4a). When pre-treated at pH 6, instead of pH 1, compounds (**1**), (**2**) and (**3**) were diminished, little affected and increased respectively (Fig. 4b). Therefore, if cmpd (**1**) was the reductant that delays peroxidase action, then its effect should be weakened by storage at pH 6.

**Fig. 5:**
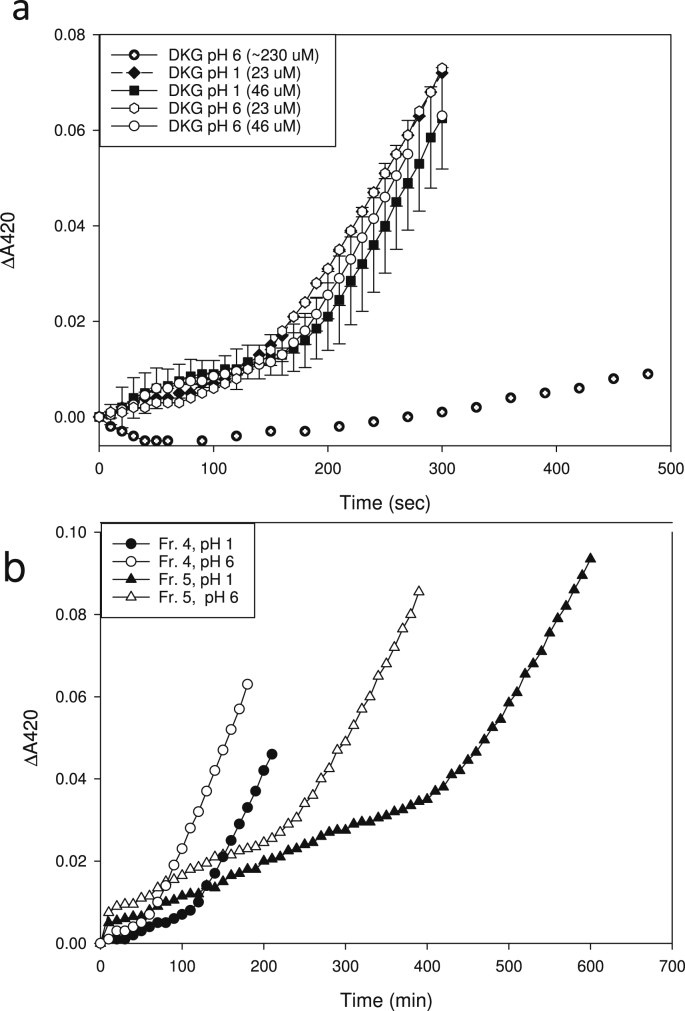
Effect of pH on the ability of diketogulonate and/or its by-products to delay peroxidase action. The samples tested were (a) the whole DKG preparation was stored at 0 °C for 0.5–3.0 h at pH ∼1 or at pH ∼6; and (b) HPLC fractions thereof that had been collected in 47 mM H_2_SO_4_ (pH ∼1) as in Fig. 2a. In each case, the samples were then tested for effect on *in-vitro* peroxidase action with ABTS as substrate. In (a), the lag times caused by the whole preparation were very long, so the samples were diluted for the assay (to ∼23 and 46 μM final). In (b), with HPLC fractions 4 and 5, undiluted 100-μl portions were included in 1-ml peroxidase reaction mixtures with Na_2_-succinate addition to buffer the pH above 5.

This prediction was tested on samples stored at pH 1 or 6 before HPLC. Pre-treatment of the unfractionated preparation only slightly diminished its ability to delay peroxidase action regardless of the pH to which they were adjusted (Fig. 5a), possibly because other reductants e.g. compound (**3**) increased after treatment at pH 6 (Fig. 4). However, in HPLC-purified preparations (fractions 4 and 5), storage at pH 6 did weaken the effect compared with storage at pH 1, approximately halving the lag period observed before peroxidase action began (Fig. 5b) [the pH of the peroxidase reactions was adjusted to >5 with the addition of Na_2_-succinate to HPLC fractions]. These data strongly support the idea that cmpd (**1**), the main 250-nm-absorbing compound in fractions 4 and 5, was the major reductant that delayed peroxidase action on its model substrate.

### 3.9 Analysis of cmpd (**1**) by high-voltage paper electrophoresis

**Fig. 6:**
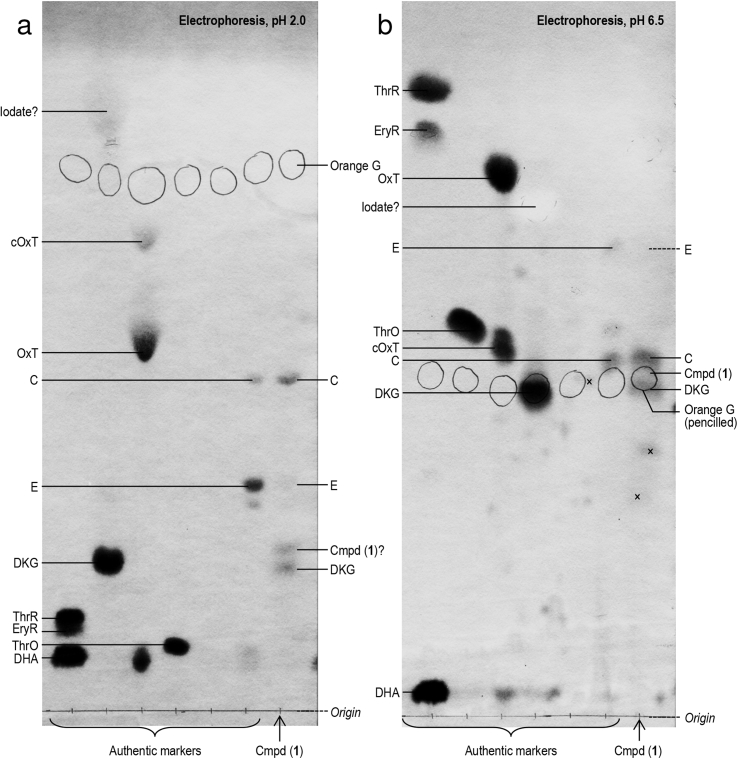
Analysis of HPLC-enriched cmpd (**1**) by high-voltage electrophoresis. Cmpd (**1**) was partially purified by HPLC with 13 mM TFA as eluent (see Supplemental Fig. 4), then analysed by electrophoresis at pH 2.0 (a) or 6.5 (b). Each sample, and the markers, contained a trace of Orange G, which was circled in pencil before the other compounds were stained in AgNO_3_. Abbreviations used: EryR, erythrarate (= *meso*-tartrate); ThrR, l-threarate (= l-tartrate); ThrO, threonate; OxT, oxalyl threonate; cOxT, cyclic oxalyl threonate; **C**, 2-carboxy-l-xylonolactone and/or 2-carboxy-l-lyxonolactone; **E**, de-lactonised **C**; DHA, dehydroascorbic acid; DKG, diketogulonate (prepared by the iodate method). Spots present in the cmpd (**1**) preparation are labelled to the right, markers to the left of each electrophoretogram. Spots labelled (×) are contaminants as they are not precisely in line with the other spots in the lane.

HVPE has proved very useful for resolving ascorbate metabolites [17], [18], [39], [38]. Partially purified cmpd (**1**) obtained by preparative HPLC with 13 mM TFA as eluent (chosen because it is readily volatile) gave stainable spots on analytical electrophoretograms (Fig. 6). The HPLC profile with TFA as eluent (Supplemental Fig. 4a) was broadly similar to that with H_2_SO_4_ (Fig. 2a), DKG eluting slightly before cmpd (**1**).

The greatest amounts of cmpd (**1**) were found between 10.5 and 11.25 min (Supplemental Fig. 4b and c). This 0.75-min window of fractions was pooled, dried *in vacuo* and re-dissolved in water; portions were electrophoresed at pH 2.0 and 6.5 and stained with AgNO_3_ (Fig. 6), revealing:

• DKG, which is only partially resolved from cmpd (**1**) by the HPLC system used;
• compounds **C** and **E** (a trace), which are proposed [39] to be **C** = 2-carboxy-l-xylonolactone plus 2-carboxy-l-lyxonolactone; **E** = their de-lactonised product (a dianionic carboxypentonate); and
• a spot whose *m*_DKG_ values were 1.22 at pH 2.0 and 1.05 at pH 6.5, which stained a slightly yellowish brown rather than the greyish brown usually produced by AgNO_3_.

**Fig. 7:**
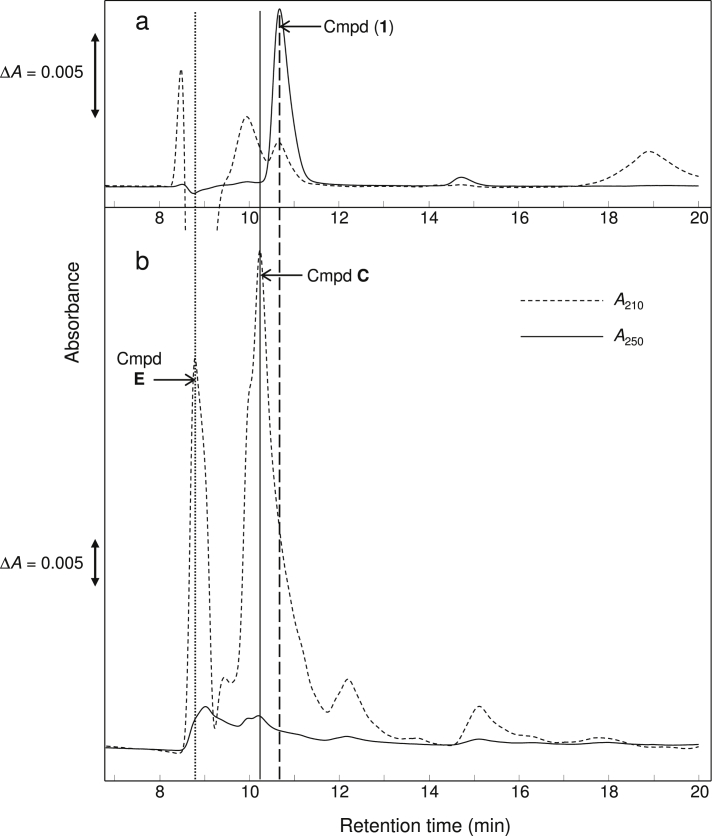
Cmpd (**1**) is not a carboxypentonate. (a) Cmpd (**1**), purified by HPLC eluted in 13 mM TFA, was re-run by HPLC in water and the products were detected by *A*_210_ and *A*_250_. (b) A sample containing compounds **C** and **E** (carboxypentonates, prepared by alkali treatment of DHA and subsequent elution from a preparative electrophoretogram) was also run by HPLC in water and monitored by *A*_210_ and *A*_250_.

Of these possible identities, we have already shown that cmpd (**1**) is not DKG itself. Furthermore, we found that cmpd (**1**) is not **C** or **E** since purified **C** and **E** gave peaks clearly resolved from cmpd (**1**) on HPLC (Fig. 7). This was demonstrated when samples of **C** and **E** (eluted from paper after preparative electrophoresis [17]; were analysed by HPLC with 13 mM TFA as eluent (Fig. 7b), and a sample containing cmpd (**1**) was run immediately thereafter (Fig. 7a). **C** and **E** showed major peaks of *A*_210_ at 10.1 and 9.2 min respectively, and only small peaks of *A*_250_ (Fig. 7b); in contrast, cmpd (**1**) showed greater absorbance at 250 nm and eluted at 10.5 min (Fig. 7a). Thus cmpd (**1**) is clearly distinguished from cmpds **C** and **E**. Compounds **C** and **E** form from DKG in aqueous conditions [17], [39], and the spots of them seen in Fig. 6 would have formed from the DKG after being eluted from the column.

We therefore suggest that, of the spots seen in Fig. 6, one with *m*_DKG_ values 1.22 and 1.05 at pH 2.0 and 6.5 respectively is likely to be cmpd (**1**). Although its structure remains unknown, some of its ionic properties can be deduced from the electrophoretic mobilities. At pH 6.5, all –COOH groups are almost fully ionised, so the compound's proximity to DKG and **C** at that pH (Fig. 6b) indicates that it has a charge:mass ratio similar to theirs, i.e. 1 negative charge per ∼6 carbon atoms. Furthermore, since the compound appears to be anionic even at pH 2.0, with a mobility exceeding that of DKG (*m*_DKG_ = 1.22; Fig. 6a), it is probably a strong acid with a p*K*_a_ even lower than that of DKG (predicted p*K*_a_ ≈ 2.38; http://www.hmdb.ca/metabolites/HMDB05971).

### 3.10 Mass spectrometry of cmpd (**1**)

A further sample of cmpd (**1**), partially purified by HPLC as in Supplemental Fig. 4, was analysed by MS in positive mode (Supplemental Fig. 5). Ion peaks at *m*/*z* 159.03164 and 181.01351 were observed, which were absent from the blank. These ions are interpreted as C_6_H_6_O_5_·H^+^ and C_6_H_6_O_5_·Na^+^ (*m*/*z* values respectively 17 and 12 ppm deviation from theoretical, which is acceptable since the nearest calibration point was at *m*/*z* ≈ 332).

## 4 Discussion

Freshly prepared DKG is reported to have little UV absorbance at wavelengths above about 225 nm [36]. However, our DKG preparations rapidly produced a proportion of compound (**1**), with *λ*_max_ 271 and 251 nm at neutral and acidic pH respectively. These *λ*_max_ values may be compared with those of ascorbate and some of its previously reported degradation products (Table 1). Cmpd (**1**)’s *λ*_max_ values, and its bathochromic shift when the pH is adjusted from acidic to neutral, suggest some chemical similarities to ascorbate. Furthermore, like ascorbate, DHA and 3,4-enediol-DKGL [48], cmpd (**1**) was more stable at acidic than neutral pH.

In agreement with the noted resemblance between cmpd (**1**) and ascorbate, the delaying effect of cmpd (**1**) on peroxidase action mimicked that of ascorbate. Ascorbate serves as an anti-oxidant, scavenging peroxidase-generated phenolic radicals, so that the oxidation of aromatic substrates becomes visible only when all ascorbate has been oxidised [50]. Thus, cmpd (**1**) probably affects peroxidase action in a similar manner.

In the present work, several metabolites obtained from a DKG preparation induced the non-enzymic production of H_2_O_2_ and delayed the onset of substrate oxidation in *in-vitro* peroxidase assays. A scheme for ascorbate degradation has been proposed [17], [18], [39], [38] in which the initial oxidation product, DHA, is either further oxidised (to oxalyl threonate, cyclic oxalyl threonate and oxalate + threonate) or hydrolysed (to DKG and its own downstream products **C** and **E**). The oxidising branch was proposed to include, or lead to, three steps that might generate H_2_O_2_. On the other hand, the hydrolytic pathway was not proposed to yield H_2_O_2_; therefore the discovery reported here that DKG by-products, principally cmpd (**1**), do generate ROS, probably mainly H_2_O_2_, was of great interest.

Cmpd (**1**) has two effects which superficially seem contradictory: (a) when present in peroxidase assays it appears to serve as an anti-oxidant, scavenging phenolic radicals such that the oxidation of aromatic substrates (*o*-dianisidine and ABTS) becomes visible only when all the cmpd (**1**) has been oxidised, and (b) it non-enzymically reduces O_2_ to H_2_O_2_, the oxidising substrate of peroxidase. Effect (a) would delay peroxidase action, whereas effect (b) would promote it. Both these effects could have biological significance in the plant cell wall. Effect (a) would delay the peroxidase-catalysed cross-linking of cell-wall phenolics [50], e.g. of ferulate to diferulates and tyrosine to isodityrosine, thus potentially preventing wall tightening. Conversely, the H_2_O_2_ generated in effect (b) can non-enzymically lead to the formation of other ROS. In particular, the hydroxyl radical (^•^OH) is readily formed from H_2_O_2_, especially in the presence of some remaining ascorbate [12], the precursor of cmpd (**1**). It is known that ^•^OH causes non-enzymic scission of cell-wall polysaccharides [12], [45], [52], potentially loosening the primary cell wall. Preventing wall tightening and promoting wall loosening, caused by (a) and (b) respectively, are both expected to lead to a more readily extensible or softer wall. Therefore the two apparently divergent effects of cmpd (**1**) may act in an equivalent direction, both of them facilitating biological processes that depend on a ‘loose’ cell wall such as cell expansion, fruit softening and abscission. Additionally, similarly to ascorbate, cmpd (**1**) may influence the oxidative burst occurring during pathogen attack, and hence plant defence responses [41].

The only known source of cmpd (**1**), potentially exerting such wall-loosening effects, is DKG — which is formed by the non-enzymic hydrolysis of apoplastic DHA. DHA can itself be formed from apoplastic ascorbate, about 50% by endogenous AAO action (which produces monodehydroascorbate followed by disproportionation), and 50% non-enzymically [18]. The ability of apoplastic AAO to generate monodehydroascorbate and hence DHA, and thus initiate the DHA → DKG → cmpd (**1**) pathway with consequent wall loosening, may explain the hitherto mysterious observation that AAO activity (which destroys ascorbate) often correlates positively with rapid plant cell growth, despite the fact that rapid growth usually correlates with high ascorbate biosynthesis [30].

Cmpd (**1**) proved highly unstable and we were unable to obtain it in a form pure enough to provide satisfactory NMR. Nevertheless, cmpd (**1**)’s early elution from a Rezex HPLC column [retention time comparable to those of DKG (Fig. 2, Fig. 4, Supplemental Fig. 4a) and oxalate (Fig. 7 of [38]] suggests that it is a relatively strong acid. Its strongly acidic nature is also indicated by the anionic spot observed on electrophoresis at pH 2.0 of a sample enriched in cmpd (**1**) (Fig. 6). This would exclude various dioxo compounds previously reported [34] as DKG degradation products such as xylosone, erythrosone, threosone, and 3-deoxyerythrosone.

MS revealed a compound with the formula C_6_H_6_O_5_, whose H^+^ and Na^+^ adducts were observed (Supplemental Fig. 5). A potential structure for C_6_H_6_O_5_ is shown in Fig. 2a. The siting of hydroxy groups and double-bonds shown is arbitrary, but this suggested lactone structure somewhat resembles ascorbic acid, which would account for cmpd (**1**)’s reducing properties and its susceptibility to ascorbate oxidase. Such a compound would be expected to ionise on the oxygen at C-3, giving an electrophoretically mobile anion. Although it remains unknown by what reaction such a structure could be formed, the process would involve the DKG (C_6_H_8_O_7_) serving as an oxidising agent (C_6_H_8_O_7_ → C_6_H_6_O_5_ + H_2_O + [O]) in a reaction in which some other compound would be oxidised by the ‘[O]’.

We conclude that cmpd (**1**) is a hitherto unreported acidic reducing agent produced by oxidation of DKG.

## 5 Conclusion

Cmpd (**1**), a product of ascorbate formed via the pathway ascorbate → dehydroascorbic acid → DKG → cmpd (**1**), delays peroxidase action *in vitro*. The build-up of cmpd (**1**) in the cell wall would decrease peroxidase-catalysed scavenging of H_2_O_2_ and thus lead to an accumulation of H_2_O_2_ into the apoplast. This, and the additional H_2_O_2_ formed non-enzymically from O_2_ by cmpd (**1**), may have profound effects on the physiology and further behaviour of the cells. H_2_O_2_ can have direct effects in the cell wall (reviewed in Ref. [23], or as a signalling molecule it can mediate changes in gene expression leading to various biological effects [57] and affect plant-pathogen interactions [41].

## Figures and Tables

**Table 1:** Ultraviolet absorption properties of ascorbate and some of its degradation products.

Compound	*λ*_max_ at acidic pH (nm)	*λ*_max_ at neutral pH (nm)	References
Cmpd (**1**)	251	271	present work
l-Ascorbate	245	265	[19]
Dehydro-l-ascorbic acid^a^	<195		[54]
	223		[2], [54], [59]
	225^w^		
2,3-Diketo-l-gulonate	<195	<225	[36], [54]
l-Erythroascorbate	245	265	[19]
2,3-Enediol-DKGL	210, 300	225, 345	[29], [51]
3,4-Enediol-DKGL	245	265	[29], [36]
2-Furoic acid	255	245	[26], [60]
	252		
5-Methyl-3,4-dihydroxytetrone	245	265	[26]
l-Erythrulose	279	279	[33]
Oxalate	<205	<205	
Succinate^b^	<205	<205	

w = Weak absorbance maximum.

a Fig. 3a of [21] shows that fresh dehydroascorbic acid has almost no absorbance at ∼300 nm, but acquires absorbance at or near that wavelength after lengthy storage of the solution.

b Not a product of ascorbate catabolism, but used in the present work as a buffer.
