# Supplementary material for: Metabolites of 2,3-diketogulonate delay peroxidase action and induce non-enzymic H2O2 generation: Potential roles in the plant cell wall
Source: Arch Biochem Biophys. 2017 Apr 15;620:12–22. doi: 10.1016/j.abb.2017.03.006 (PMC5398285; doi:10.1016/j.abb.2017.03.006)
Supplement: Supplementary Figs [file mmc1.pdf]

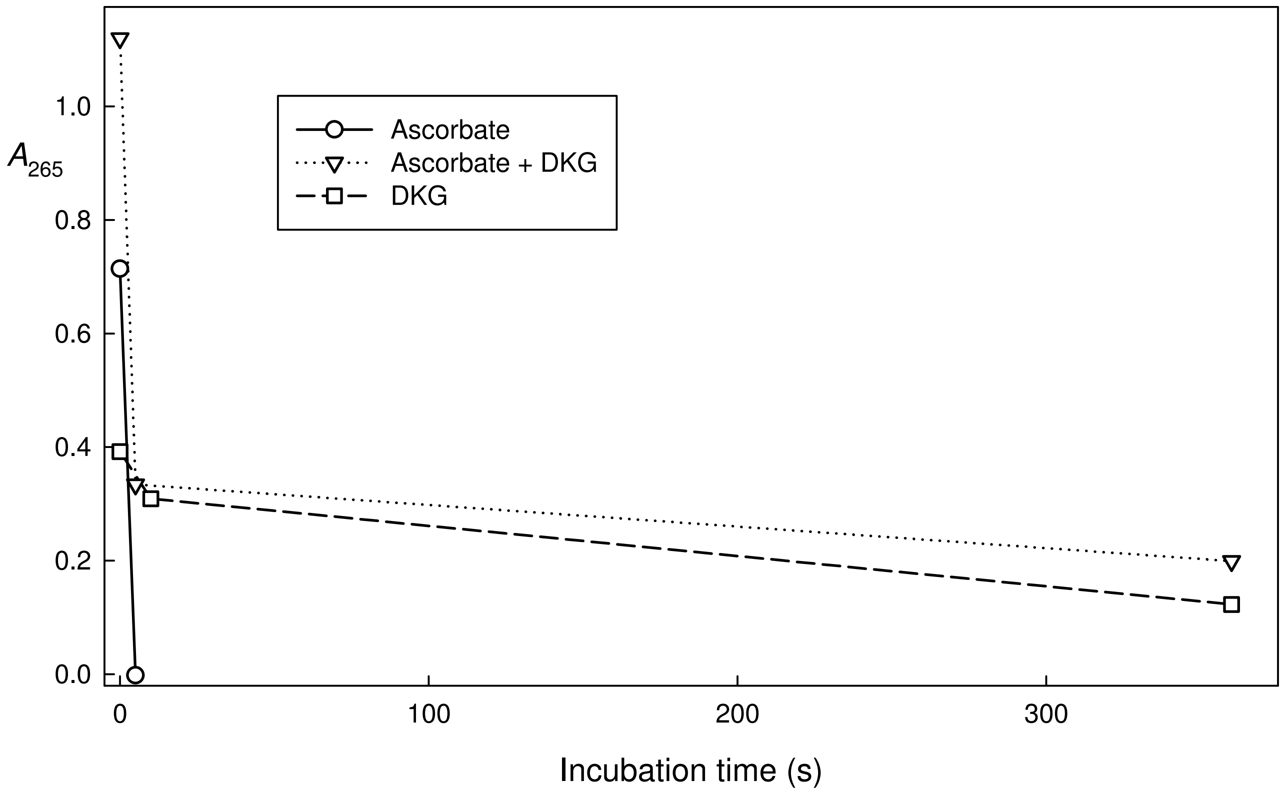

Supplemental Fig. 1. Effect of ascorbate oxidase on ascorbate, diketogulonate by-products, and a mixture of ascorbate and diketogulonate by-products.
Ascorbate oxidase (AAO, final concentration 4 U/ml) was added to cuvettes containing 50 µM ascorbate, ~460 µM of a diketogulonate (DKG) preparation, or a mixture of 50 µM ascorbate and ~460 µM of the DKG, each in 50 mM succinate (Na^+), pH 5.6.

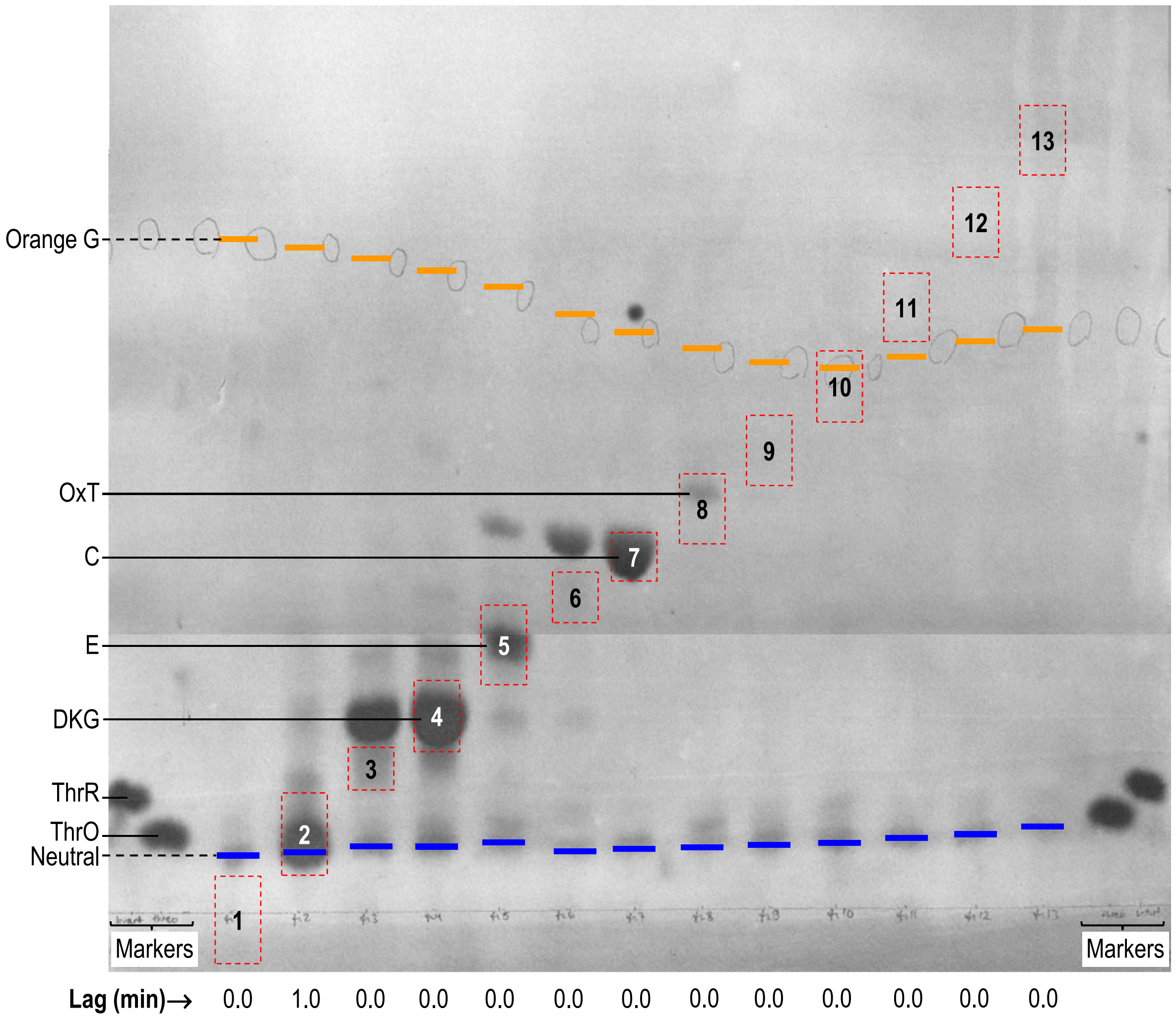

Supplemental Fig. 2. Analytical electrophoretograms of fractions eluted from preparative electrophoretograms of diketogulonate and its by-products. (a) Electrophoresis at pH 2.0. The crude DKG preparation was subjected to preparative electrophoresis at pH 2.0, then thirteen (unequal) fractions were eluted: fr1 was the material that remained close to the origin; fr2 contained neutral material (co-migrating with glucose, which moves slightly from the origin owing to electro-endo-osmosis) plus weak acids that are only slightly ionised at pH 2.0 (represented by the threonate marker); .....; and fr13 was the fastest migrating towards the anode. A small portion of each eluted fraction was then re-run by *analytical* electrophoresis at pH 2.0 (3.5 kV, 30 min) and stained with $AgNO_3$ (shown here). The red boxes indicate the approximate positions on the *preparative* electrophoretogram from which each fraction had been eluted, these positions being adjusted with respect to the reference compounds glucose (m_{OG} = 0.00; blue bar) and Orange G (m_{OG} = 1.00; orange bar). Additional portions of each fraction were concentrated and tested for their ability to delay peroxidase action on *o*-dianisidine (producing the lag times indicated). The marker, Orange G (pencilled spot), was loaded *between* each sample. '**C**' and '**E**' are DKG breakdown products provisionally identified as carboxypentonates, **C** being a lactone of **E**. Abbreviations: ThrO, L-threonate; ThrR, L-threarate (= L-tartrate); OxT, oxalyl threonate.

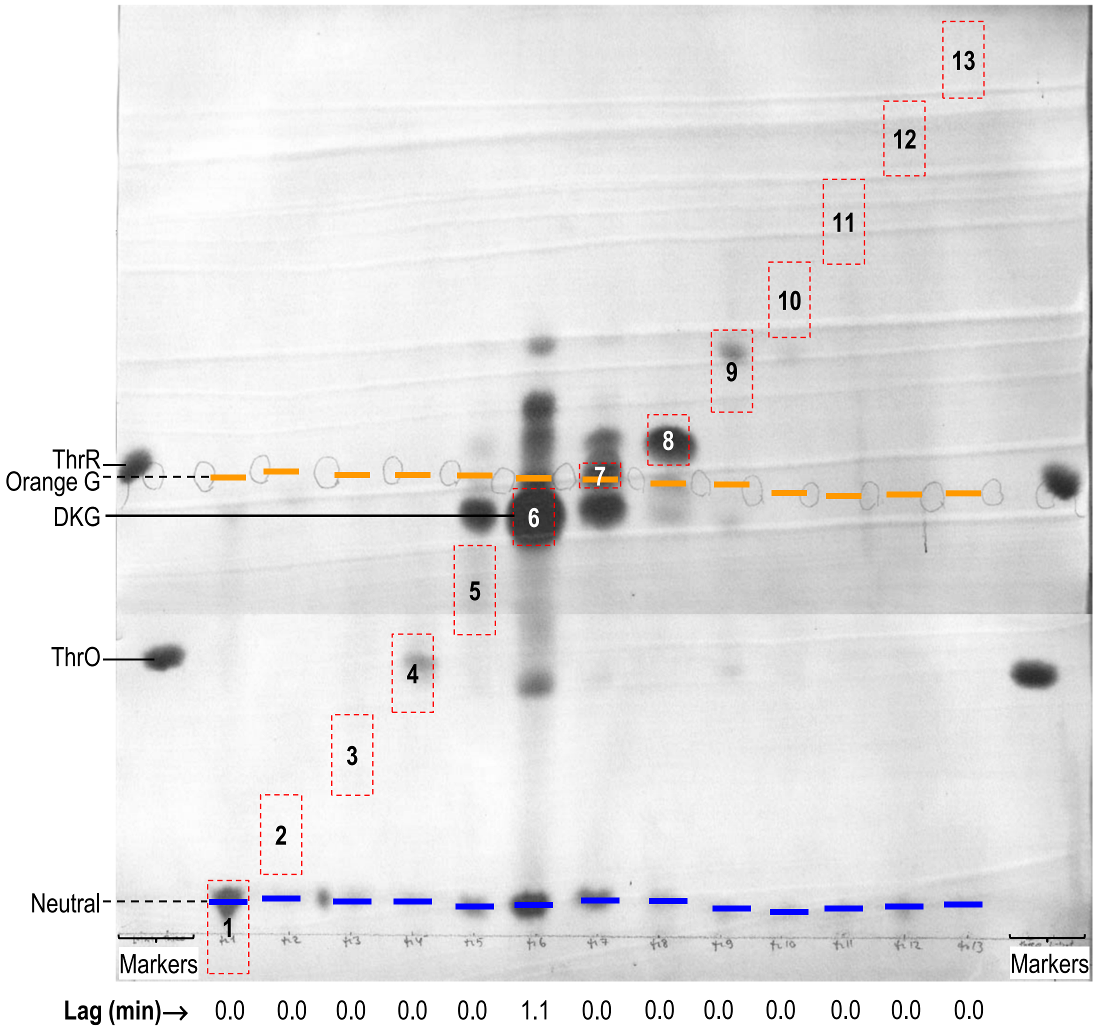

Supplemental Fig. 2. Analytical electrophoretograms of fractions eluted from preparative electrophoretograms of diketogulonate and its by-products. (b) Electrophoresis at pH 3.5. Details as in (a), but both the preparative (not shown) and the analytical electrophoretograms (3.5 kV, 30 min; shown here) were run in pH 3.5 buffer.

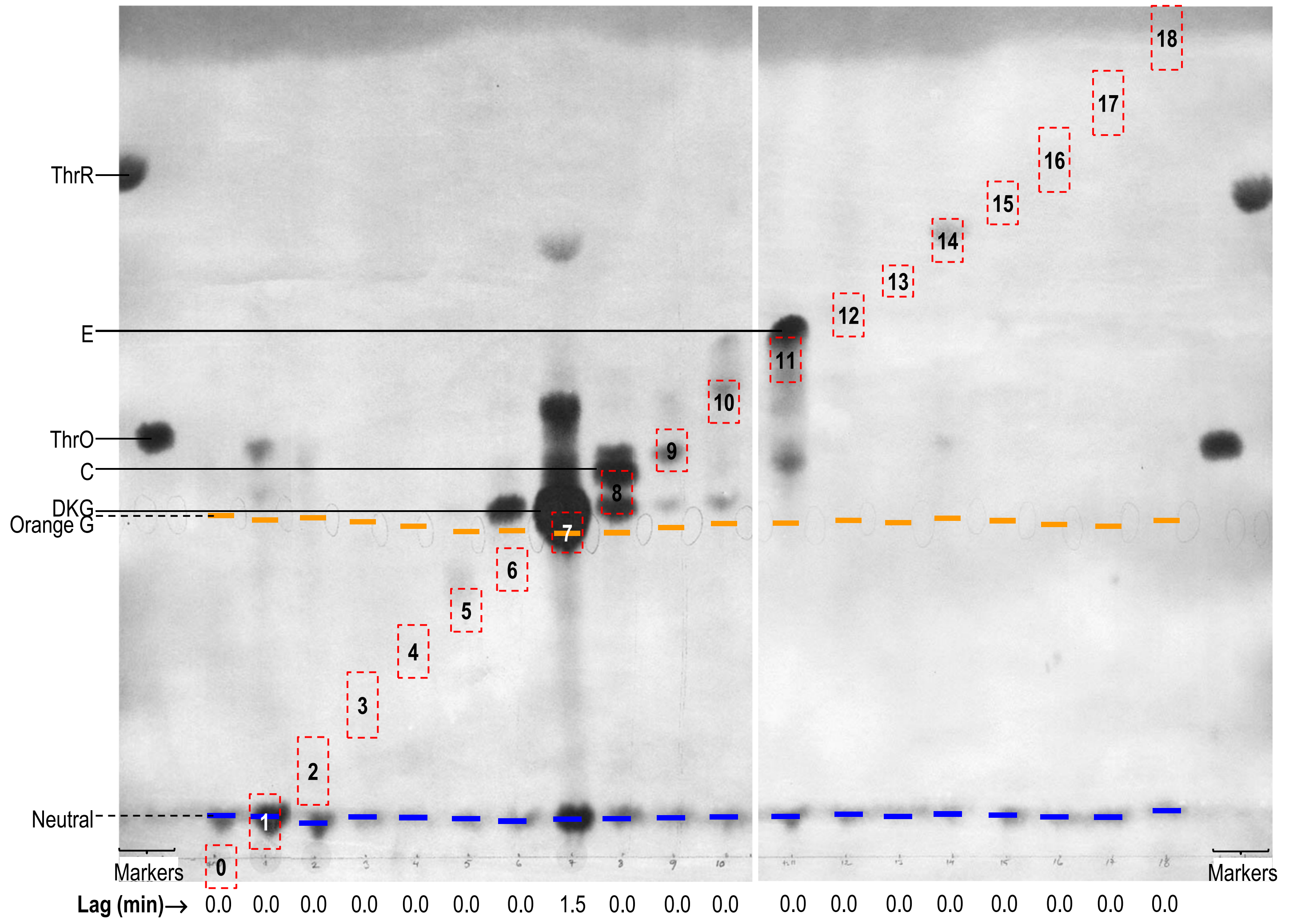

Supplemental Fig. 2. Analytical electrophoretograms of fractions eluted from preparative electrophoretograms of diketogulonate and its by-products. (c) Electrophoresis at pH 6.5. Details as in (a), but both the preparative (not shown) and the analytical electrophoretograms (3.5 kV, 30 min; shown here) were run in pH 6.5 buffer.

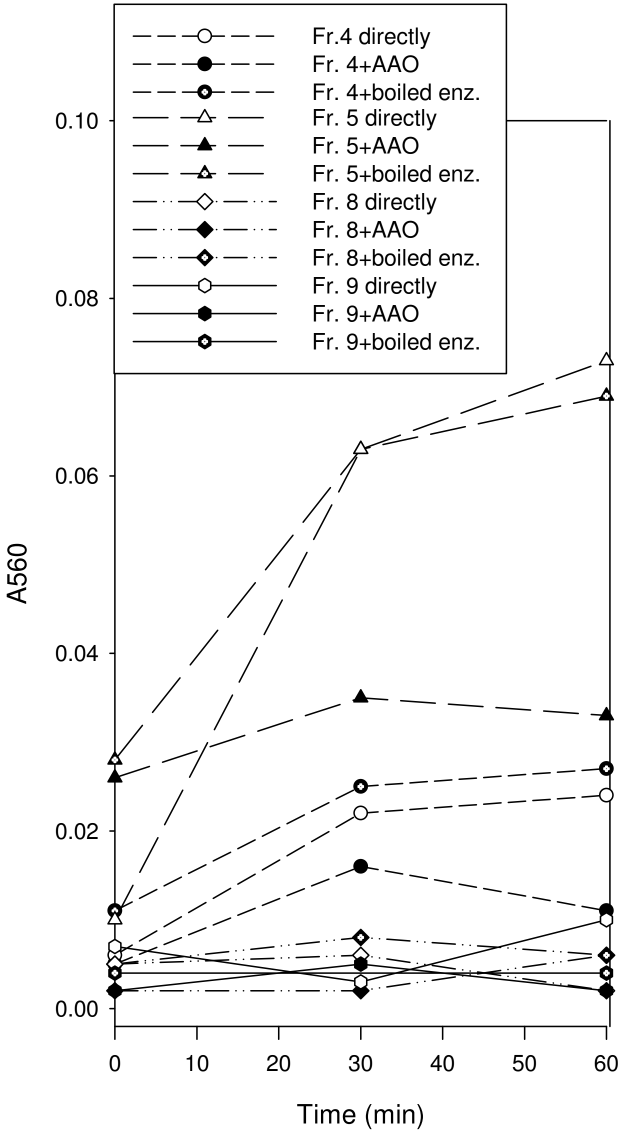

Supplemental Fig. 3. Non-enzymic generation of H_2O_2 by selected HPLC fractions from the diketogulonate preparation. HPLC fractions 1–13 were collected as shown in Fig. 2, treated for 10 min with or without fresh ascorbate oxidase (AAO, 2 U per 3 ml of the reaction mixture composed of 150 µl fraction, 75 µl 47 mM H_2SO_4 and 8.33 mM Na_2-succinate; final pH 5–6) or with heat-denatured AAO, then assayed for H_2O_2 production. The reaction was initiated by addition of 1 µM $CuSO_4$. A solution of 1 µM $CuSO_4$ was used as a blank for the XO assay and therefore gave A_{560} = 0.

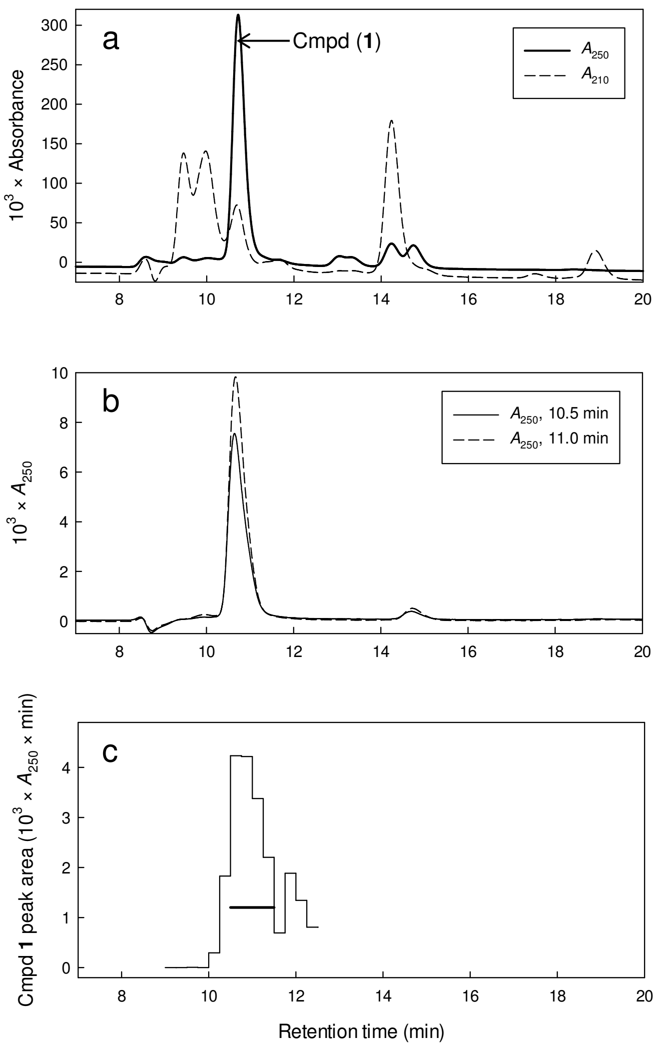

Supplemental Fig. 4. Purification of cmpd (**1**) by preparative HPLC.
(a) DKG was aged at 0°C for 24 h, then run by HPLC in water. Absorbance was measured at 210 and 250 nm simultaneously. (b) Fractions (each 15 s) were collected, then 25-µl aliquots were re-run by HPLC with 13 mM TFA [0.1% (v/v)] as eluent; two examples are shown here. The A_{210} cannot be shown because TFA absorbs strongly at that wavelength. (c) From the areas of these peaks, the yield of cmpd (**1**) in each fraction, judged by A_{250}, was reconstructed. The 10.50–11.25-min fractions (black bar) were pooled for use in electrophoresis.

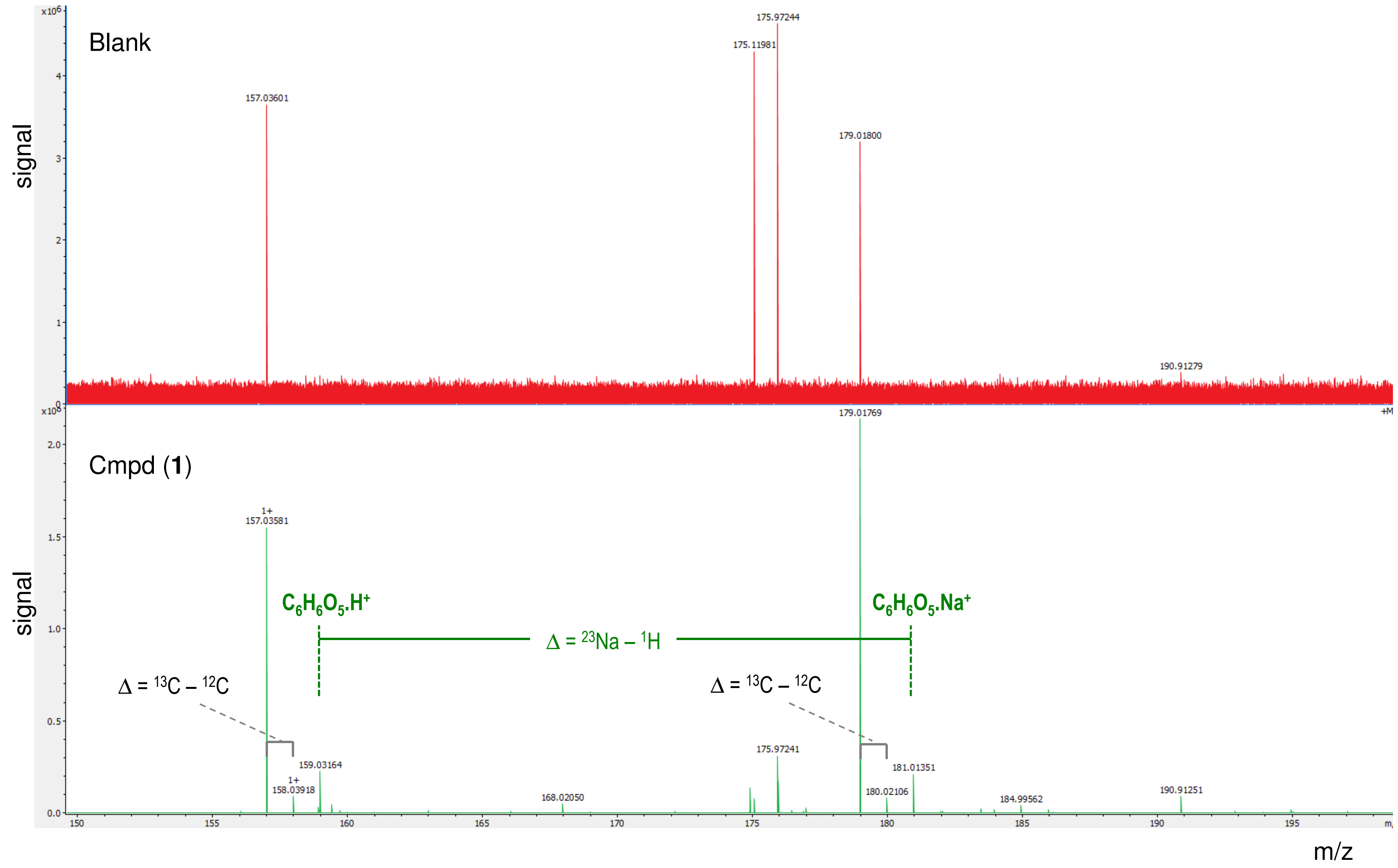

Supplemental Fig. 5. Mass spectrometry of HPLC-purified cmpd (**1**).
Above: control without ascorbate-related material present.
Below: MS of HPLC-purified cmpd (**1**). The ions with m/z 158.03918 and 180.02106 are interpreted as the same as those at 157.03581 and 179.01769 but with one ^{13}C atom.
